# Comparing the Response of Birds and Butterflies to Vegetation-Based Mountain Ecotones Using Boundary Detection Approaches

**DOI:** 10.1371/journal.pone.0058229

**Published:** 2013-03-11

**Authors:** Rafi Kent, Oded Levanoni, Eran Banker, Guy Pe’er, Salit Kark

**Affiliations:** 1 The Biodiversity Research Group, Department of Ecology, Evolution and Behavior, The Hebrew University of Jerusalem, Jerusalem, Israel; 2 ARC Centre of Excellence for Environmental Decisions, School of Biological Sciences, The University of Queensland, Brisbane, Queensland, Australia; University of Guelph, Canada

## Abstract

Mountains provide an opportunity to examine changes in biodiversity across environmental gradients and areas of transition (ecotones). Mountain ecotones separate vegetation belts. Here, we aimed to examine whether transition areas for birds and butterflies spatially correspond with ecotones between three previously described altitudinal vegetation belts on Mt. Hermon, northern Israel. These include the Mediterranean Maquis, xero-montane open forest and Tragacanthic mountain steppe vegetation belts. We sampled the abundance of bird and butterfly species in 34 sampling locations along an elevational gradient between 500 and 2200 m. We applied wombling, a boundary-detection technique, which detects rapid changes in a continuous variable, in order to locate the transition areas for bird and butterfly communities and compare the location of these areas with the location of vegetation belts as described in earlier studies of Mt. Hermon. We found some correspondence between the areas of transition of both bird and butterfly communities and the ecotones between vegetation belts. For birds and butterflies, important transitions occurred at the lower vegetation ecotone between Mediterranean maquis and the xero-montane open forest vegetation belts, and between the xero-montane open forest and the mountain steppe Tragacanthic belts. While patterns of species turnover with elevation were similar for birds and butterflies, the change in species richness and diversity with elevation differed substantially between the two taxa. Birds and butterflies responded quite similarly to the elevational gradient and to the shift between vegetation belts in terms of species turnover rates. While the mechanisms generating these patterns may differ, the resulting areas of peak turnover in species show correspondence among three different taxa (plants, birds and butterflies).

## Introduction

Areas of ecological transition, or ecotones, are related to sharp environmental gradients and are often characterized by high species turnover rates and local biodiversity peaks [1], [2], [3]. As such, they are considered important from both a theoretical point of view (e.g., for studying community species assembly rules), and an applied perspective, e.g., in selection of areas for conservation or as indicators of climate change [4], [5], [6], [7]. For instance, many studies have shown that mountain treelines exhibit a response to increase in temperature and therefore the location of the treeline may be used as an indicator of global warming [8], [9], [10]. The concept of ecotones was first used to describe abrupt changes in species composition related to habitat structure [2]. Examples include bird communities at the interface between forest and grassland [3], [11], [12] and alterations of vegetation formation [5]. Ecotones also occur at diverse spatial scales [5], and may vary in width, from narrow with sharp transition rate to wide with moderate transition rate [3]. Many taxonomic groups exhibit transitional areas separating adjacent communities [2], [13].

Boundary detection techniques enable to perform spatially explicit analyses where one or more variables, either environmental (e.g., altitude or primary productivity) or biological (e.g., species abundances), are assessed to identify turnover rates of that variable(s), and assign a location according to the rate of change. Locations with relatively high turnover rates are then considered as boundary elements [14]. Detection of boundaries depends on the measure of biodiversity used in the analysis [6]. For example, if two sites have similar species richness then a boundary would only be detectable when using data on abundances, and if they have the same diversities as well then only data on species composition could be used [15], as was done in this study.

Mountains are highly suitable for ecotone studies, due to the sharp gradients occurring over small geographic extents [16], [17]. Sharp gradients have several advantages for research due to these characteristics for example: (i) species turnover rates are potentially high in various groups, following the high rates of environmental change; and (ii) the small geographic extents covered by the local gradient minimize the effects of historical and evolutionary processes that might confound the ability to distinguish ecological processes.

Birds and butterflies have been shown to exhibit similar responses to environmental change in previous studies [18], [19], including along elevation gradients [7]. An open question remains, however, whether different taxonomic groups, in our case birds and butterflies, have spatially congruent transition areas within the same domain (along the same environmental gradient, such as elevation) and how changes in birds and butterflies are related to ecotones of vascular plants. Such congruence may suggest that the environmental gradient affects different taxonomic groups in a similar manner, at least with regards to species turnover rates. Alternately, transition areas of different taxonomic groups may differ in their spatial location due to the perception of environment by different organisms at different spatial scales [20].

Due to the direct and indirect dependence of both birds and butterflies on vegetation, both on its physiognomy and species composition, we anticipated both butterflies and birds to follow changes in vegetation formation and thus to show spatially congruent boundaries with those described for the vegetation on Mt. Hermon in previous studies [21], [22]. The underlying mechanisms of that spatial congruence between boundaries of birds and butterflies and those of plants may differ. For example, many butterfly species are dependent on their host plant species, which respond to vegetation zonation [23], [24], though the rate of change of this response may differ from that of the change in vegetation structure. Breeding birds are generally less dependent on specific plant species than butterflies, but depend on vegetation structure for shelter, foraging, nesting and social activities [25]. Thus one could predict that birds will exhibit clear boundaries, around the same elevations as the transitions in vegetation belts. In this study we ask whether butterfly and bird communities exhibit similar spatial patterns in response to changes in vegetation along a sharp elevational gradient on Mt. Hermon, Northern Israel. Butterflies can be affected by elevational gradients [26], [27]. Elevation effect on butterflies has been attributed to the correlation between elevation and climatic conditions, such as temperature and water availability and changes in vegetation [26], [28]. In addition, many butterfly species are host-specific, i.e. they require the presence of a specific plant species to complete their life cycle e.g., [23]. As plants also react strongly to environmental variation that is affected by elevation, the response exhibited by butterflies to elevation might be spatially correlated to that of plants. Responses of bird communities to elevational gradients often involve decline in species richness with increasing elevation or unimodal patterns, and have been attributed to reduction in forested area and a decline in abundance and size of invertebrates [29], [30], [31]. We aimed to examine whether the spatial location of transition areas was congruent across the elevational gradient, and whether they spatially correspond with the boundaries between vegetation belts (Mediterranean maquis, xero-montane open forest and mountain steppe Tragacanthic vegetation).

## Methods

### Ethics Statement

All necessary permits were obtained for the described field studies. The permit was issued by the Israel Nature and Parks Authority (permit number: 2005/21852).

### Study Area

**Figure 1:**
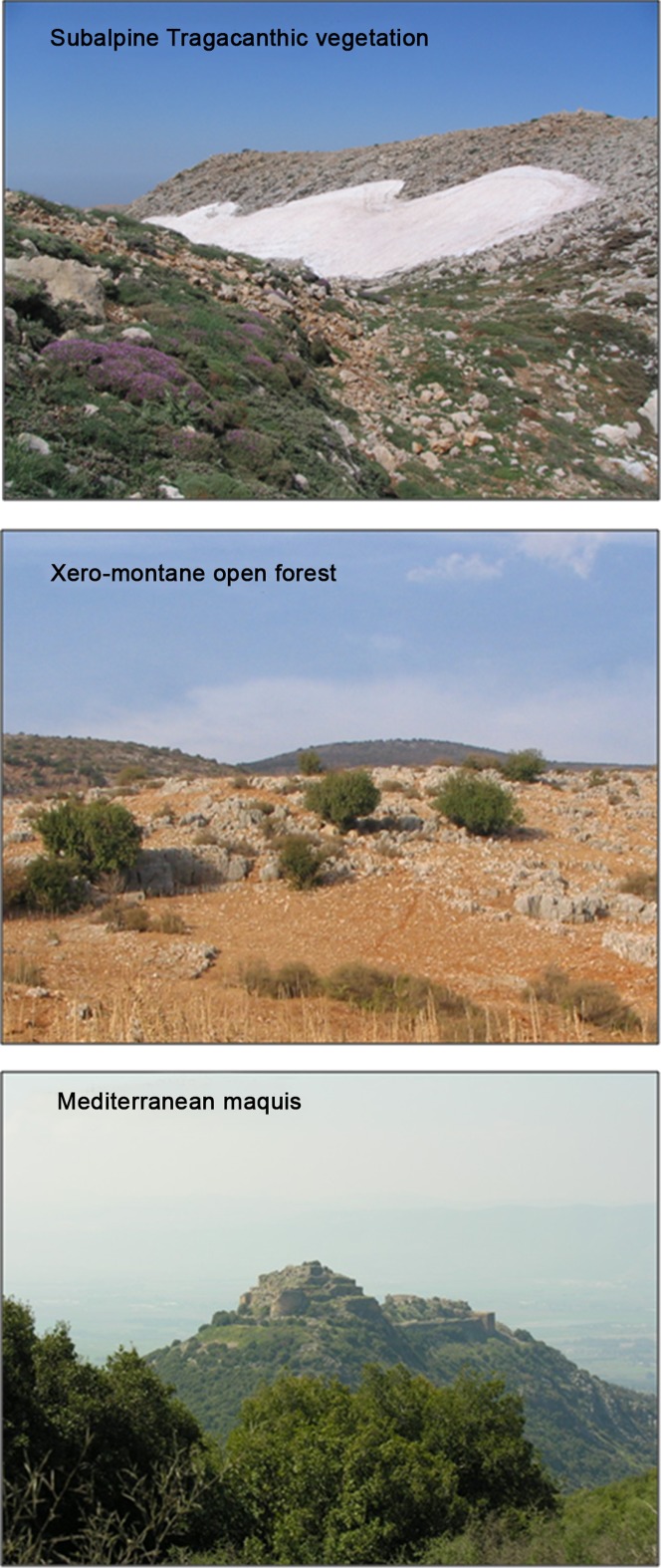
Representative vegetation in the three vegetation belts along the elevation gradient in the Mt. Hermon study area. Photos are by Salit Kark (bottom and top) and Oded Levanoni (middle).

The study took place along an elevational gradient on Mt. Hermon, Northern Israel (33°25′N, 35°48′E), near the border with Lebanon and Syria (Fig. 1). The Hermon is an elongated anticline extending NE-SW over 35 km and covers an elevation range from 300 to 2,814 m ASL. The Israeli Hermon ranges ca. 7,300 ha, and covers a 13-km distance on the SW tip of the mountain range. The highest point in the Israeli part of the Hermon is at 2,224 m. Most of the area is a nature reserve since 1972 [21]. The parent material is homogeneous, hard Jurassic limestone [21], [32]. The terrain of Mt. Hermon is characterized by steep rocky slopes. Its climate is Mediterranean, with rainy, cold winters and hot, dry summers. Precipitation ranges from 600 to 1500 mm per year, and above 1500 m consists mostly of snow [22]. During summer and autumn, snow patches remain only at elevations above 1900 m. Three main vegetation belts have been described in earlier studies of Mt. Hermon [21], [22], [33] (Fig. 1). These include: (i) evergreen Mediterranean maquis (300–1,250 m); (ii) xero-montane open forest (1250–1850 m) and (iii) subalpine mountain steppe, or “Tragacanthic belt” (1,850–2,814 m) [22].

### Sampling Design

In order to select study sites that are most representative of their elevation belt, we calculated the average NDVI values of each 50 m elevation belt in the whole study area, and preselected the sampling site within each belt in a location with NDVI value as close as possible to the average in that elevation belt [21], [23]. Further details about the pre-selection method of sampling sites can be found in Levin et al. [21]. We sampled birds and butterflies in 34 locations along the elevational gradient on the southwestern slope of the mountain, between 500 and 2200 m ASL. Data on butterflies and birds from the exact same location was collected for 31 of the 34 locations, and thus three locations were omitted from the comparative analyses (omitted sampling locations were at 1,030 m, 1,490 m and 1980 m). Sampling sites were pre-selected to minimize the variability caused by soil type, slope, aspect, vegetation cover. All locations were located on a SW-facing aspect, had a slope steepness of 30–60%, tree cover below 50% and rock cover under 30%. We maximized distance from anthropogenic influence (i.e. roads and settlements) in all sampling locations.

### Butterfly Sampling

Butterfly sampling was conducted over two years during the peak activity season of most Hermon butterflies (February-September 2005 and March-August 2006; *sensu* Benyamini [34], [35]). Butterfly sampling was conducted by two Lepidoptera experts capable of recognizing all species in the field (O.L. and G.P.). A butterfly expert and a note-taker walked along parallel lines inside each sampling 50×20 m quadrat for 20 minutes. The sampling duration of 20 min in the quadrats was determined based on species-accumulation curves generated in preliminary work (see [34] for details) using *Estimate*S Version 8.0 [36]. This preliminary work indicated that an asymptote in species richness is reached, on average, after 12±3 minutes of sampling (sampling ≥95% of the species).

### Bird Sampling

Birds were sampled by an experienced birder (E.B.) using point sampling [37]. Sampling points were located within the same quadrats where butterfly sampling took place. Bird sampling was conducted during the breeding season, between March and August 2005 and between April and July 2006. Bird sampling was performed by allowing a 5-min acclimation period upon arrival at the sampling site, and then counting all birds seen or heard within a 200 m radius during a 10 min sampling period. Sampling duration was determined by preliminary samplings. To avoid biases associated with visiting sites in the same sequence, travel routes were alternated between visits [34]. Our full dataset included all species observed, including migrant species that stopped-over but did not breed. As the bird community in the study is comprised of both non-breeding (transient migratory) species, and breeding (resident species and summer-visiting) species, we also used a subset that consisted of observations of breeding species, to isolate any possible effect of migratory state on the location of ecotones along the elevational gradient. Individuals that flew over during the count but were not directly interacting with the environment were omitted from the analyses.

Detectability is dependent on both time of sampling and species. To ensure that detectability differences did not affect our findings, we used a preliminary estimation of the species richness in each station for the same datasets, which was performed using mark-recapture bootstrapping techniques (Turbe et al., unpublished data). The estimations revealed a correlation of 0.85 between observed and expected species richness. Therefore, we used the original raw data, with all visits pooled in our analyses.

### Boundary Detection

In order to detect transition areas representing locations with high species turnover rates, we used the boundary detection software BoundarySeer 1.0 [14]. We used the wombling technique for vector point data to detect boundaries in species composition, and applied it to species abundance data [14]. Wombling is a term describing the use of Womble’s edge detection algorithm to calculate the magnitude of the rate of change in spatial data [12], [38]. Results are probabilistic. The area among the sampling locations is divided into potential boundary elements, and the probability of each potential boundary element is returned. Boundary elements are determined as the elements with the highest probability to constitute a boundary [14], [21]. As change rates along elevational gradients are often gradual, this tool allowed us to systematically locate areas with relatively high change rates. Each selected boundary element (out of the potential elements detected in the wombling process) was assigned a geographical location. We recorded the elevation of each boundary element using GIS. We also related each boundary element to the nearest sampling station in our dataset.

Results of the boundary detection analyses were compared to the location of vegetation belts, as derived from Levin et al. [21], Shmida [22] and A. Shmida (personal communication). One boundary that was detected at the elevation of 900 m was suspect to be biased due to its higher proximity to human settlement (∼180 m from the settlement border, compared to the next nearest site that was ∼360 m from any settlement) In order to examine whether the boundary signal around 900 m is an outlier we omitted that sampling location from the analyses and repeated it again. If this omission would still reveal a boundary between neighboring stations then the removed station would be considered as a true boundary. However, if an analysis without the 900 m station would have revealed no boundary around that elevation, then this location could be considered an outlier.

## Results

### Butterfly Boundaries Along the Elevation Gradient

**Figure 2:**
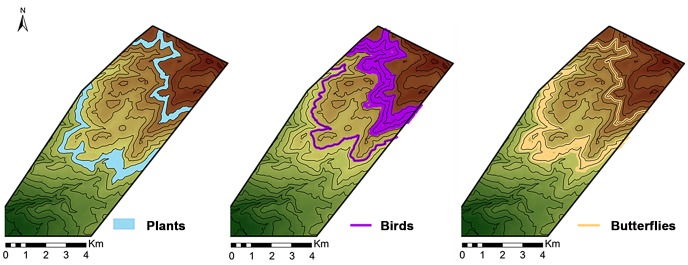
A graphic representation of the ecotones detected in the study site (for birds and butterflies in purple and orange respectively) compared with vegetation belts, as described in previous studies of Mt. Hermon (Shmida 1974). Black contours denote 100 m elevation intervals. The background color gradient represents elevation from low (in green) to high (in brown).

**Figure 3:**
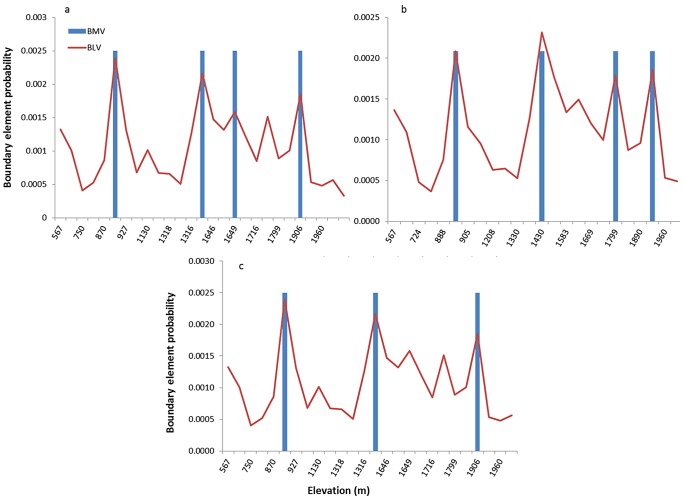
Boundary likelihood values of the different elevations along the sampled transect (lines), along with the detected boundaries, presented as BMV (Boundary Membership Value), a dichotomous variable, where 1 denotes boundary membership and 0 indicates no membership (blue bars). Results are shown for a) butterflies, b) all bird species, and c) breeding bird species only. Note that in all groups, the boundary element around 900 m was found to be an outlier.

A total of 76 butterfly species belonging to six butterfly families were sampled within the 31 sampling locations included in the analysis. Boundary detection analysis using the wombling technique [14] showed three transition areas for butterflies. The highest probability for the existence of a boundary was detected between 1275–1400 m ASL (Fig. 2). This transition area is spatially adjacent to the boundary between Mediterranean Maquis and xero-montane open forest, located at 1200–1300 m [32]. A second boundary was detected around 1800 m (Fig. 2), congruent with the upper plant ecotone (at 1800–1900 m). The likelihood of each station to be considered a boundary element in the wombling process is presented in Fig. 3a. An additional sampling location with relatively high rate of species turnover was found around 900 m. Due to topographic constraints, the sampling locations around this elevation were located closer to human disturbance than other points, 180 meters from the village of Neve Ativ. Probability of detected boundary elements was about double that of the background value (Fig. 3).

Butterfly species richness exhibited a unimodal response to elevation (the quadratic term of the relationship was y = −0.11+0.029x−8.67*10^−6^x^2^, linear and quadratic terms had P-values of 0.006 and 0.02 respectively, Mitchell-Olds Shaw test indicated a significant hump-shaped curve) while species diversity, as measured by the Shannon-Wiener index, was significantly and positively correlated to elevation (Pearson’s r = 0.54).

### Bird Boundaries Along the Elevational Gradient

Boundary detection analyses using the wombling method revealed two transition areas in the bird community along the elevational gradient (Fig. 2). Both complete and breeding species analyses revealed a boundary at an elevation of 1400 m, indicating high rates of change in community composition at this elevation. A second boundary was detected between 1800 and 1900 m for the entire species pool, while breeding species had a more diffuse boundary around the same elevations, between 1650 and 1900 m (Figure 3b,c). Probability of detected boundary elements was nearly double that of the background value (Fig. 3). Similar to the results for butterflies, there was a signal of a boundary at 900 m ASL, near the village of Neve Ativ. We repeated the omission of the location at 900 m in order to examine whether it is an outlier. After omission no boundary was detected for birds around 900 m. An outlier analysis revealed that a boundary detected around 900 m elevation was likely an artifact caused by proximity to human settlement.

Bird species richness was linearly and negatively correlated with elevation (Pearson’s correlation coefficient r = 0.7 and r = 0.72 for the entire species pool and for breeding species respectively). Shannon-Wiener diversity was strongly and negatively correlated with elevation (Pearson’s r = −0.84) for both the entire species pool and the breeding species.

## Discussion

High mountains are often composed of vegetation belts that are defined based on differences in vegetation forms and communities among different belts [39], [40]. Located at the meeting area between Africa, Europe and Asia, a meeting area of biogeographic regions, Mt. Hermon provides a useful case study for examining shifts in communities among vegetation belts [22], [33]. The vegetation on Mt. Hermon has been divided into three vegetation belts [21], [22]. This segregation into belts corresponds strongly with the elevational gradient of ∼2500 m over a span of 13 km [21]. Differences between the vegetation belts do not constitute separate plant communities [32], but mostly depict a significant change in vegetation formation (Fig. 1). Our results reveal that both butterflies and birds respond similarly to the elevational gradient and to the shift between vegetation belts. By contrast, species richness and diversity in these two taxa respond differently to the elevational gradient. The lower transition area of the butterfly community, as defined using boundary detection, was detected between 1,275 and 1,400 m ASL, while the vegetation transition was between 1200–1300 m. The discrepancy between the shift in vegetation formation and the boundary detected in the butterfly community may be caused by the high mobility of butterflies, allowing individuals to be active beyond the border of the vegetation belt. Analysis of the bird data revealed a boundary in the composition of the community at 1400 m, suggesting that the transition from Mediterranean Maquis to xero-montane open forest has significant effects on the bird community.

A second transition area, at higher altitude, was detected for both birds and butterflies occurring at 1800–1900 m. However, butterflies exhibited a more abrupt change, leading to the detection of a boundary, while birds exhibited a transition area that varied in width between 100 to 250 m of elevation range, depending on the species included in the analyses (the whole dataset or breeding species only). The relatively low probability values of the detected boundaries (Fig. 3) are likely attributed to the large number of species in the study area. Previous studies that used wombling techniques and reported higher probabilities focused on a single species [41] or type of disease [42] as the subject of analysis.

The differences found between groups may be explained by different mechanisms causing the spatial response of the two groups to the elevational gradient. We hypothesize that while the high turnover in butterfly species is caused by physiological constraints related to elevation, the dependency of birds on vegetation belts may be related to the structure of the vegetation in the area. Birds use plants for shelter, foraging, as breeding sites and in their social behaviors [31]. Different bird species have different adaptations to habitat structure, which might lead to the observed pattern of dependency of birds on vegetation belts and ultimately elevation. The analysis of both taxonomic groups showed an additional, relatively high probability of a third boundary, near the village of Neve Ativ, around 900 m. The proximity to the village suggests that there is an anthropogenic effect around that elevation, increasing species abundances, and attracting commensal species, resulting in an outlier, rather than an effect of the elevation or other environmental gradients. This assumption was corroborated by further analyses, excluding the 900 m sampling location, in which no boundary was detected between 800 and 1000 m elevations, as expected if a true boundary existed. In addition to the proximity to Neve Ativ, we believe the butterfly community might have been affected by the hilltop locality and the complex architecture of the location. This is because many butterfly species congregate on hilltops for the purpose of mating [43], [44], [45].

Similar spatial patterns of variability in the community composition of two taxonomic groups as distant as birds and butterflies may indicate that the effect of elevation is not group specific, but rather a common phenomenon [46]. The results of this study conform to the theoretical model proposed by Terborgh [31], [47]. While Terborgh did not find that ecotones explained most variance in his system, he suggested that ecotones explain a relatively large proportion of the variance in species distribution patterns at the community level, in communities that are distributed along gradients that include a sharp environmental change in habitat. Our results support the existence of transition areas in both bird and butterfly communities, related to the structurally variable vegetation belts at 1200–1300 m and 1800–1900 m.

Although the rates of change in species turnover along elevational gradient are gradual and the wombling approaches quantify relative changes, the Boundary Detection tools allow us to elegantly and quantitatively identify locations with changes in species composition, which have important ecological implications. For example, in Mt Hermon, we find along the gradual elevational gradient steep and rapid exchange of the three vegetation belts [22]. While the structural vegetation transition among these belts is rapid, the change in plant community composition is much slower [32]. However, we find it interesting that both birds and butterflies also show peaks in turnover rates in elevations that are close to those where the major transition between vegetation belts occur. It remains to be tested in future studies how each species responds to structural changes along the elevational gradient.

Detection of transition areas has been proposed as a useful tool for monitoring changes in distribution in response to climate change [10]. It has been found that birds and butterflies, as well as other taxa, are extending their distributions northwards and upwards [48]. Our results imply that northwards and upwards shifts across transition areas may be detectable at local spatial scales in mountains. Increasing the efficiency of transition area detection by fine-scale field sampling, monitoring and computational tools, can be further applied in spatial conservation planning and management.
